# Supplementary material for: Decreased continuous sitting time increases heart rate variability in patients with cardiovascular risk factors
Source: PLoS One. 2021 Jun 16;16(6):e0253399. doi: 10.1371/journal.pone.0253399 (PMC8208552; doi:10.1371/journal.pone.0253399)
Supplement: S1 Table — (PDF) [file pone.0253399.s001.pdf]

ID	Dcrease or Increase	sex	age	BMI	Baseline				After 6 months			
					Sitting time	Sleep time	Physical activity	LogHF	Sitting time	Sleep time	Physical activity	LogHF
1	Increase	male	76	21	65	540	0.53	2.56	87	480	0.56	2.54
2	Decrease	male	79	23	65	360	0.56	2.12	49	360	0.67	2.12
3	Decrease	male	69	25	65	600	0.55	2.24	60	480	0.59	2.31
4	Decrease	male	76	18	65	420	0.52	2.26	29	540	0.72	2.35
5	Decrease	male	70	21	95	480	0.99	2.42	0	480	1.05	2.46
6	Decrease	Female	60	24	50	480	0.86	1.89	0	420	1.02	2.19
7	Increase	male	74	23	75	480	0.96	2.76	80	540	1.02	2.73
8	Decrease	Female	83	20	85	480	0.63	2.27	60	480	0.5	2.44
9	Decrease	male	79	25	40	600	0.41	2.28	0	480	0.46	2.5
10	Increase	Female	59	25	0	540	0.54	2.32	108	480	0.67	2.26
11	Increase	male	81	26	80	620	0.78	2.88	136	322	0.81	2.36
12	Increase	male	61	22	60	420	1.37	2.06	60	480	1.34	2.08
13	Decrease	male	74	19	55	540	0.52	2.43	39	480	0.44	1.99
14	Increase	Female	71	29	40	420	0.61	2.6	68	480	0.72	2.48
15	Increase	Female	68	23	0	540	0.76	2.53	0	480	0.81	2.24
16	Increase	Female	65	24	40	480	0.68	2.05	55	480	0.73	2.08
17	Increase	male	69	24	40	540	0.75	2.42	78	540	0.83	2.41
18	Decrease	male	71	25	70	420	0.91	2.6	0	420	0.85	2.64
19	Increase	male	77	24	115	540	0.74	2.34	115	540	0.58	2.63
20	Decrease	Female	76	37	115	480	0.73	2.3	35	480	0.62	2.18
21	Increase	Female	79	20	30	720	0.48	2.14	40	540	0.76	2.17
22	Increase	male	73	20	65	540	0.54	1.85	85	480	0.49	1.69
23	Increase	male	77	22	75	420	0.5	1.71	90	420	0.64	2.16
24	Increase	Female	69	22	0	430	0.71	1.76	80	430	0.81	1.75
25	Increase	male	67	27	0	480	1.03	2.13	0	480	1.07	2.28
26	Increase	male	69	21	0	300	0.76	2.36	50	300	0.88	2.4
27	Decrease	male	61	22	50	420	1.11	2.17	0	540	1.03	2.51
28	Increase	Female	76	23	0	540	0.65	1.99	70	540	0.81	2.23
29	Increase	Female	67	23	0	600	0.92	2.43	0	540	0.88	2.62
30	Increase	male	80	23	0	480	0.94	2.13	0	420	0.82	2.29
31	Decrease	Female	67	28	35	420	0.66	2.37	0	480	0.66	2.43
32	Increase	Female	66	31	0	360	0.75	1.83	0	420	0.82	1.85
33	Increase	male	69	35	55	540	1.16	1.97	60	540	1.09	1.74
34	Increase	Female	67	29	0	420	0.89	1.9	0	420	0.93	2.13
35	Increase	male	67	26	0	540	1.18	1.92	0	480	0.95	2.29
36	Decrease	male	68	25	55	420	1.46	2.06	50	480	1.35	1.96
37	Increase	male	73	26	55	420	1.11	1.73	60	420	1.07	1.64
38	Increase	Female	74	19	0	480	0.86	2.41	0	420	0.71	2.47
39	Decrease	male	77	21	60	500	0.54	2.25	25	505	1.25	2.15
40	Increase	Female	81	24	65	556	0.6	1.2	105	530	0.65	1.3
41	Increase	Female	73	24	0	521	0.51	1.53	0	450	0.48	1.48
42	Decrease	male	78	19	60	470	0.48	1.99	0	500	0.48	2.08
43	Decrease	male	80	23	60	300	0.4	2.26	0	390	0.62	2.05
44	Increase	male	75	23	0	605	0.97	2.91	40	505	1.09	2.88
45	Decrease	male	76	27	60	365	0.57	2.41	40	480	0.92	2.27
46	Decrease	male	79	23	90	578	0.82	2.16	65	470	0.78	2.54
47	Decrease	Female	73	23	40	465	0.62	2.69	30	395	0.64	2.8
48	Decrease	Female	75	29	70	470	0.66	2.82	30	415	0.73	3.09
49	Decrease	male	79	22	135	539	0.69	2.29	0	505	0.95	2.34
50	Increase	male	72	26	60	395	0.71	2.06	75	395	0.79	2.16
51	Decrease	Female	60	26	120	290	0.75	2.4	45	445	0.66	2.66
52	Increase	Female	85	17	70	455	0.43	2.03	70	430	0.42	1.7
53	Increase	Female	72	20	0	510	0.86	1.95	70	455	0.76	1.94
