# Supplementary material for: Decreased continuous sitting time increases heart rate variability in patients with cardiovascular risk factors
Source: PLoS One. 2021 Jun 16;16(6):e0253399. doi: 10.1371/journal.pone.0253399 (PMC8208552; doi:10.1371/journal.pone.0253399)
Supplement: Attachment — Submitted filename: Revise 20210513.docx [file pone.0253399.s002.docx]

|  | Reviewer's Comment | Author's Response |
| --- | --- | --- |
|  |  | We wish to express our sincere appreciation to the reviewers for their insightful comments on our paper. The comments have helped us significantly in improving our paper. |
| 1 | Please provide a sample size and power calculation in the Methods, or discuss the reasons for not performing one before study initiation. | Since this study was a post hoc analysis, we did not calculate the sample size or the detection power before study initiation. |
| 2 | Please amend your current ethics statement to include the full name of the ethics committee/institutional review board(s) that approved your specific study.  Once you have amended this/these statement(s) in the Methods section of the manuscript, please add the same text to the “Ethics Statement” field of the submission form (via “Edit Submission”). | Thank you for your precise comments. We have fixed it as follows: The present study was conducted with the approval of the Bioethics Review Committee at Nagoya University (260024-1, 201700953). |
| 3 | a. Please provide an amended statement that declares *all* the funding or sources of support (whether external or internal to your organization) received during this study, as detailed online in our guide for authors at http://journals.plos.org/plosone/s/submit-now  Please also include the statement “There was no additional external funding received for this study.” in your updated Funding Statement.  b. Please include your amended Funding Statement within your cover letter. We will change the online submission form on your behalf. | We appreciate your thoughtful comments. We have added: There was no additional external funding received for this study.  In addition, we have added the same sentence to the cover letter. |
| 4 | Please include captions for your Supporting Information files at the end of your manuscript, and update any in-text citations to match accordingly. | Thank you for your precise and professional  comments. We added supporting information. |
|  | Reviewer 1 |  |
| 1 | There was no mention on echocardiographic assessment for the patients included, which I think is relevant to the outcomes for these patients. | We appreciate your thoughtful and constructive suggestions. We agree with you. Because patient symptoms were mild and well controlled by medication as outpatients and because echocardiography was not necessarily performed periodically due to the reduced possibility of remarkable differences in LV mass index and LV wall motions, we did not utilize echocardiographic data for the present study. Thank you again for your informative suggestions. We would like to consider echocardiographic data for a future study. |
| 2 | The discussion part is deviated from the main theme of the study, please elaborate more about the results you obtained in the experiment ie(HF HRV and change in sitting behavior) | Thank you for your helpful suggestions. We have added a discussion of sitting behavior with HF HRV and modified some of the text structure to further convey the results of the present study. |
|  | Reviewer 2 |  |
| 1 | The authors should provider how they arrived at the sample (53) of participants that participated in their study. Were the participants selected at random? | Thank you for highlighting this point. We have revised the text as follows: In the present study, all patients provided complete details on their lifestyles and had visited outpatient clinics for 6 consecutive months (n = 53). Participants had no cardiovascular events such as myocardial infarction or stroke before December 2019． |
| 2 | Under the Ethical consideration (page 7, lines 123-127) the authors need to specify the institutions that granted the ethical approval(s) for their study and the corresponding reference numbers. The authors are affiliated to five (5) institutions and this needs to be specified. | Thank you for your precise comment. We have revised the text as follows: The present study was conducted with the approval of the Bioethics Review Committee in Nagoya University (260024-1, 201700953).　Because the authors now work for 5 institutions and are employees, graduates, or honorary professors at Nagoya University and because the analyses of the study were mainly performed at Nagoya University, the ethical approval was obtained in Nagoya University on behalf of the affiliated institutions. |
| 3 | On page 8, line 151, the authors should replace the word “deceased” with “decreased”. | We appreciate your helpful and precise suggestion. We have revised it as correctly suggested. |
| 4 | On page 13, the authors should consider providing a limitation of the methods used in their study. The authors also mentioned on lines 215 - 216 that “Patients who participated in this trial suffered from mild hypertension and/or stable angina pectoris.” The authors need to specify whether or not they conducted a trial and if so which type? In addition, was the trial registered? and was the protocol number provided anywhere in the methods? | Thank you for your correct and informative comment. This study was a post hoc analysis and was not registered as a prospective investigation. We removed the unnecessary words and have modified the text as follows: Patients who participated in this analysis had mild hypertension and/or stable angina pectoris. |
